# An Interesting Anomalous Coronary Artery: Right Coronary Artery Arising from the Mid Part of the Left Anterior Descending Artery

**Published:** 2017-04

**Authors:** Allahyar Golabchi, Ali Abbasi, Mohamad Ramezani

**Affiliations:** 1 Faculty of Medicine, Kashan University of Medical Sciences, Kashan, Iran.; 2 Shaheed Beheshti Hospital, Kashan University of Medical Sciences, Kashan, Iran.

**Keywords:** *Coronary vessels*, *Coronary vessel anomalies*, *Coronary angiography*

A 48-year-old woman was admitted to the emergency department with complaints of typical chest pain, cold sweat, and dyspnea of 24 hours’ duration. She had a history of hypertension, hyperlipidemia, and diabetes mellitus for many years. On admission, her electrocardiogram (ECG) revealed normal sinus rhythm and dynamic T inversion in the precordial leads. Her enzyme levels were normal. Transthoracic echocardiography showed a left ventricular ejection fraction of 60%, with mild mitral regurgitation. Because of ST depression during the peak exercise test, cardiac catheterization was suggested and then performed. Coronary angiography demonstrated normal left main coronary artery courses with normal dominant left circumflex artery and left anterior descending artery (LAD). An anomalous right coronary artery (RCA) was seen, with a nondominant separate branch arising from the mid part of the LAD. It then coursed anteriorly down on the right atrioventricular groove (Figure 1). Attempts for the cannulation of the RCA were unsuccessful. Aortic root angiography did not show the presence of an independent-origin RCA from the ascending aorta (Figure 2 and Figure 3). We selected medical management of X syndrome (microvascular disease) and followed up the patient because of the nondominancy of the RCA. There was no need for revascularization or surgery. She was discharged in good condition.

**Figure 1:**
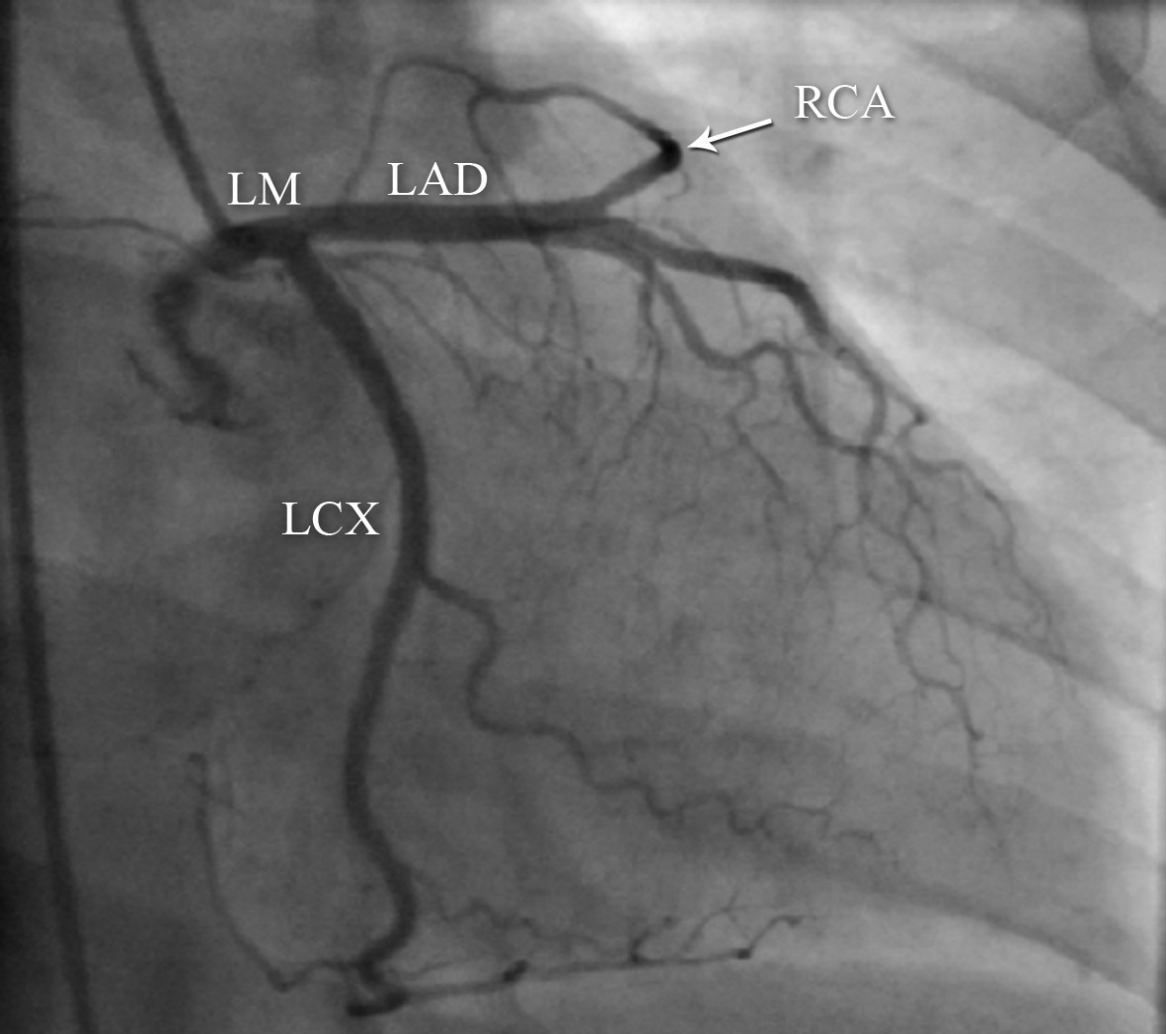
Right anterior oblique coronary angiography shows an anomalous right coronary artery (RCA), having originated from the mid part of the left anterior descending coronary artery (LAD).

LCX, Left circumflex coronary artery; LM, Left main coronary artery

**Figure 2:**
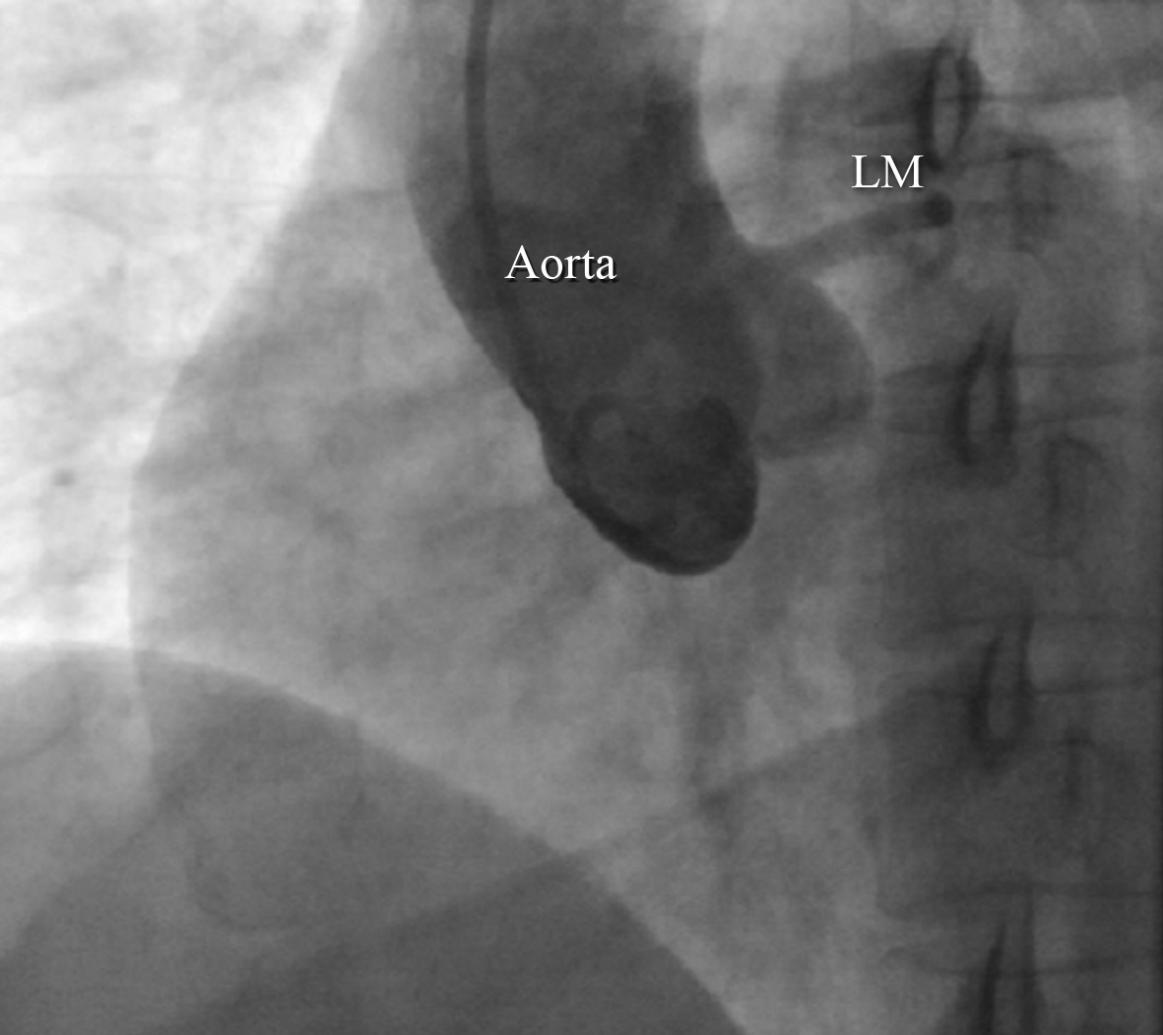
Right anterior oblique aortic root angiogram shows no presence of an independent origin of the right coronary artery.

LM, Left main coronary artery

**Figure 3:**
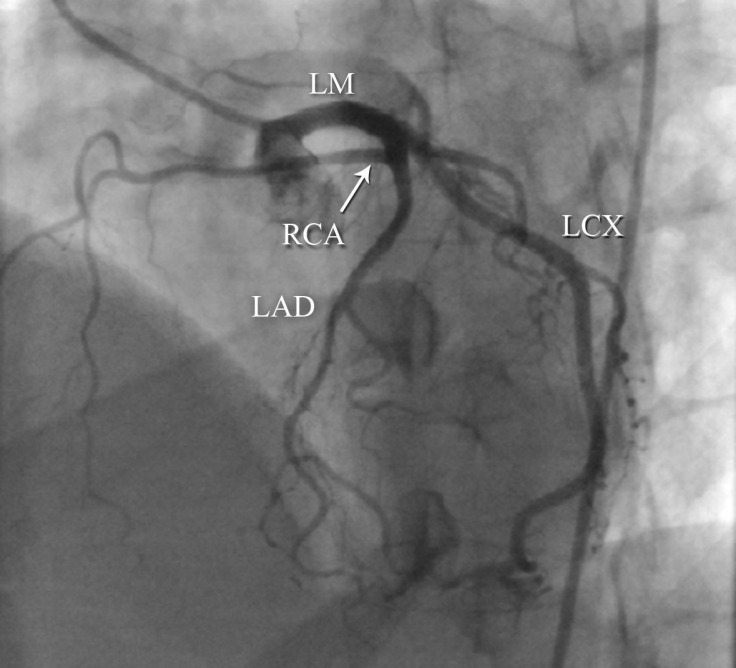
Left anterior oblique view of coronary angiography shows an anomalous right coronary artery (RCA), having originated from the mid part of the left anterior descending coronary artery (LAD).

LCX, Left circumflex coronary artery; LM, Left main coronary artery

The prevalence of the anomalous origin of the RCA from the left system varies from 0.1% to 0.9%.^1^ The risk of sudden death and the anomalous origin of the RCA from the left side has yet to be fully elucidated. Some studies have shown that the anomalous origin of the RCA is generally asymptomatic.^2^ The management of patients is different, depending on their clinical presentations, anatomical details, and additional findings.^3^

## Notes:

***This Photo Clinic should be cited as:*** Golabchi A, Abbasi A, Ramezani M. An Interesting Anomalous Coronary Artery: Right Coronary Artery Arising from the Mid Part of the Left Anterior Descending Artery. J Teh Univ Heart Ctr 2017;12(2):99-100.
